# 驱动基因阴性非小细胞肺癌二线治疗中国专家共识

**DOI:** 10.3779/j.issn.1009-3419.2024.102.10

**Published:** 2024-02-20

**Authors:** Expert Committee on Non-small Cell Lung Cancer of the Chinese Society of Clinical Oncology

**Keywords:** 肺肿瘤, 免疫治疗, 抗血管生成药物, 驱动基因阴性, 专家共识, Lung neoplasms, Immunotherapy, Anti-angiogenic drugs, Negative driver gene mutations, Expert consensus

## Abstract

对于驱动基因阴性的晚期非小细胞肺癌（non-small cell lung cancer, NSCLC）患者而言，既往化疗一直都是标准治疗选择，而免疫检查点抑制剂（immune checkpoint inhibitors, ICIs）的加入为这部分患者提供了新的治疗选择。目前一线治疗可以选择化疗、抗血管生成药物或免疫治疗。尽管初始治疗能获得一定的有效率，但仍不可避免地会出现疾病进展或治疗失败，二线及以上治疗疗效差，患者预后不佳，临床上需要更多有效的二线治疗药物。中国临床肿瘤学会非小细胞肺癌专家委员会组织呼吸科、肿瘤科、病理科专家对驱动基因阴性人群临床研究证据进行了深入探讨，根据专家组讨论后广泛认可的临床诊疗经验，对驱动基因阴性NSCLC患者二线治疗制定了统一的专家共识，可作为中国临床医师选择驱动基因阴性NSCLC二线治疗的指导依据。

## Abstract (en)

For advanced non-small cell lung cancer (NSCLC) patients with negative driver gene mutations, chemotherapy has always been the standard treatment option, and immune checkpoint inhibitors (ICIs) provide other treatment option for these patients. At present, the first-line treatment can choose chemotherapy, anti-angiogenic drugs or immunotherapy. Although the initial treatment can achieve a certain clinical curative effect, disease progression or treatment failure is eventually unavoidable. The second-line and subsequent treatments have poor efficacy and more effective drugs are needed clinically. An expert panel of respiratory medicine, pathology and medical oncology organized by Expert Committee on Non-small Cell Lung Cancer of the Chinese Society of Clinical Oncology conducted an in-depth discussion on evidences of clinical studies for second-line treatment of NSCLC patients with negative driver gene mutations, aiming to provide guidances for Chinese clinicians in choosing second-line treatment for NSCLC patients with negative driver gene mutations.

肺癌为世界发病率第二、中国发病率第一的恶性肿瘤。2016年我国肺癌新发病例高达82.81万，死亡病例高达65.7万^[1]^。非小细胞肺癌（non-small cell lung cancer, NSCLC）是肺癌的主要病理亚型，占所有病例的85%-90%^[2]^。大多数肺癌患者初次诊断时已经处于晚期。免疫检查点抑制剂（immune checkpoint inhibitors, ICIs）的治疗为驱动基因阴性NSCLC患者带来了明显获益，然而多数患者仍面临耐药问题。

NSCLC二线治疗是指在一线治疗后，患者出现疾病进展，需要启动的不同于一线方案的治疗策略。此时需要将原方案停用并更换为其他全身治疗方案。二线治疗适用于术后复发、局部晚期和晚期NSCLC首次全身抗肿瘤治疗后出现疾病进展的患者。

目前中国治疗指南中NSCLC二线治疗推荐是基于一线含铂化疗的证据，主要以单药化疗或单药免疫治疗为主，缺乏免疫经治这部分人群的研究数据，并且治疗的选择非常有限。随着一线治疗ICIs应用的增加，探索前沿治疗策略受到更多的关注。中国临床肿瘤学会非小细胞肺癌专家委员会牵头，组织相关领域专家，结合2023年版中国临床肿瘤学会指南，并参考最新国内外文献、临床研究数据及最新大会报道，在专家组共同讨论的基础上，统一意见后制定本共识，以期能为驱动基因阴性NSCLC患者的二线治疗提供参考。

## 1 方法学

本共识由中国临床肿瘤学会非小细胞肺癌专家委员会发起，专家组及执笔专家根据现有的循证学依据和临床经验，对驱动基因阴性NSCLC患者二线治疗方案进行了梳理与筛选，广泛征集多学科专家意见，最终由核心专家组讨论定稿。

根据筛选出的临床问题，检索PubMed[检索式：(((Non-small cell lung cancer[Title]) OR (NSCLC[Title])) AND ((relapsed[Title]) OR (refractory[Title]) OR (second-line[Title/Abstract]))) NOT (first line[Title]) NOT (third line[Title])]、EMBASE[检索式：'non small cell lung cancer':ti AND 'second line':ab,ti NOT 'first line':ab,ti NOT 'third line':ab,ti]、中国知网（CNKI）[检索式：(主题=肺癌) AND (关键词=非小细胞肺癌) AND (篇关摘=二线)]等数据库，采用该检索式，共检索出4801篇文献。去除重复文献172篇；限定发表时间为2013年1月1日至2023年6月30日，剔除1086篇；剔除系统综述、荟萃分析和随机对照临床研究以外文献2957篇；剔除学位论文、未发表文献239篇；剔除北大核心以外的中文文献89篇，剩余258篇文献。在此基础上，对题目、摘要（前言）、关键词等进行逐一研读，排除驱动基因阳性文献168篇，纳入90篇文献进行分析，最终选择疗效佳和安全可控的研究。

证据等级及共识度依据中国临床肿瘤学会诊疗指南，见表1。

**表1:** 中国临床肿瘤学会诊疗指南证据类别

级别	水平	说明	专家共识度
1A类证据	高	严谨的meta分析、大型随机对照研究	一致共识
1B类证据	高	严谨的meta分析、大型随机对照研究	基本一致共识，且争议小
2A类证据	稍低	一般质量的meta分析、小型随机对照研究、设计良好的大型回顾性研究、 病例-对照研究	一致共识
2B类证据	稍低	一般质量的meta分析、小型随机对照研究、设计良好的大型回顾性研究、 病例-对照研究	基本一致共识，且争议小
3类证据	低	非对照的单臂临床研究、病例报告、专家观点	无共识，且争议大

## 2 治疗前评估

驱动基因阴性NSCLC二线治疗前需要再次进行个体化诊断评估，以确定二线治疗方案的选择，主要包括基础问诊、辅助影像学检查、组织学或者细胞学检查、血清学实验室检查和病理学评估。治疗前应了解患者年龄、美国东部肿瘤协作组体能状态（Eastern Cooperative Oncology Group performance status, ECOG PS）评分、吸烟史、手术史、既往史、重要脏器功能、伴随疾病及自身免疫相关疾病、肺癌相关症状及其转移部位等，根据这些指标进行总体评估，确定患者能否耐受二线治疗。二线治疗方案的选择，需要根据一线治疗的反应、是否接受过免疫治疗、一线治疗的时间、是否存在自身免疫疾病等综合评定。

## 3 驱动基因阴性NSCLC二线治疗药物临床应用推荐

驱动基因阴性NSCLC二线治疗选择需结合一线治疗方案（是否使用抗肿血管生成药物、ICIs等）及其疗效，选择合适的治疗方案（图1）。

**图1:**
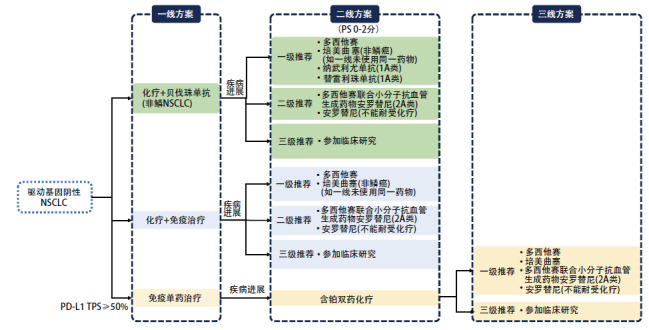
驱动基因阴性NSCLC治疗药物临床应用推荐

本共识以一线是否使用ICIs为依据，结合现有证据为患者进行临床推荐。有关非免疫经治患者目前的临床证据，已经有部分ICIs在国内获批适应证，同时，多种联合治疗方案的临床研究正在探索中，如化疗联合抗血管生成药物或抗血管生成药物联合ICIs及其他联合等。

### 3.1 非免疫经治患者二线治疗临床证据和推荐意见

**Table:** 

分层	一级推荐	二级推荐	三级推荐
PS 0-2分	多西他赛； 培美曲塞（非鳞癌） （如一线未使用同一药物）； 纳武利尤单抗 （1A类）^[3,4]^； 替雷利珠单抗 （1A类）^[5]^	多西他赛联合小分子抗血管生成药物安罗替尼（2A类）^[6,7]^； 安罗替尼（不能耐受化疗）	参加临床研究

注：针对驱动基因阴性NSCLC二线治疗，部分II期研究正在探索中并已取得初步疗效，可供临床选择：贝伐珠单抗联合多西他赛（非鳞癌）^[8]^、雷莫西尤单抗联合多西他赛^[9]^、尼达尼布联合多西他赛^[10]^。

#### 3.1.1 化疗或化疗联合抗血管生成药物

对于PS评分0-2分患者一线非免疫治疗进展后，可给予二线化疗。二线化疗中双药化疗方案较单药化疗未显示生存获益，故推荐多西他赛或培美曲塞单药治疗。不能耐受化疗或不愿意接受化疗并且PS评分>2分患者可以考虑使用小分子抗血管生成药物安罗替尼。对于PS评分>2分的患者，不建议化疗，建议最佳支持治疗。

抗血管生成药物联合化疗在欧洲已经是标准治疗，国内尚无标准，但是相关探索正在进行中。化疗联合抗血管生成药物方面，在NCT03726736/ALTER-L016研究^[6]^中，57例患者接受安罗替尼联合多西他赛治疗，31例患者接受多西他赛治疗，在至少接受1个周期治疗的患者中，与多西他赛单药比较，安罗替尼联合多西他赛延长了晚期NSCLC患者的中位无进展生存期（progression-free survival, PFS）（5.4 vs 2.4个月，HR=0.41，95%CI：0.24-0.71，P=0.001），并且提高了客观缓解率（objective response rate, ORR）（30.4% vs 14.3%）和疾病控制率（disease control rate, DCR）（96.4% vs 64.3%）。另一项NCT036243093/ALTER-L018研究^[7]^也显示安罗替尼联合多西他赛用于表皮生长因子受体（epidermal growth factor receptor, EGFR）野生型难治性晚期NSCLC患者二线治疗有获益，主要终点PFS显著延长（4.36 vs 1.64个月，HR=0.38，95%CI：0.22-0.65，P<0.001），与多西他赛单药相比，安罗替尼联合多西他赛可显著提高ORR（35.14% vs 9.52%, P=0.007）及DCR（83.78% vs 54.76%, P=0.006）。目前这两项研究总生存数据尚未成熟。考虑上述两项研究纳入患者为中国人群，药物可及性好，本共识将安罗替尼联合多西他赛作为二级推荐。

WJOG 5910L研究^[8]^中，一线贝伐珠单抗联合化疗治疗进展后的晚期非鳞NSCLC患者，100例患者随机分为贝伐珠单抗联合多西他赛组和多西他赛组，结果显示，贝伐珠单抗联合多西他赛组中位PFS为4.4个月，多西他赛组为3.4个月（P=0.058），而中位总生存期（overall survival, OS）分别为13.1 vs 11.0个月（P=0.11）。REVEL研究^[9]^为一项多西他赛联合雷莫西尤单抗二线治疗NSCLC患者的多中心、随机对照大型III期研究，共纳入1253例IV期NSCLC患者，一线化疗经治后进行1:1随机分组：多西他赛联合雷莫西尤单抗组及对照组（多西他赛联合安慰剂），结果显示，多西他赛联合雷莫西尤单抗组和对照组比较，PFS（4.5 vs 3.0个月）及OS（10.5 vs 9.1个月）均有所改善，两组患者的不良反应发生率无明显差异，通过减量或支持治疗，不良反应可控。LUME-Lung 1研究^[10]^纳入1314例化疗经治IIIB/IV期NSCLC患者，随机分为多西他赛联合尼达尼布组和多西他赛联合安慰剂组，结果显示，多西他赛联合尼达尼布组中位PFS为3.4个月，多西他赛联合安慰剂组为2.7个月，差异具有统计学意义（HR=0.79, 95%CI: 0.68-0.92, P=0.0019）；两组OS间无显著差异。而亚组分析提示，多西他赛联合尼达尼布组中腺癌患者OS为12.6个月，较对照组的10.3个月有明显提高。基于此项研究，欧洲药品管理局（European Medicines Agency, EMA）已批准多西他赛联合尼达尼布用于晚期肺腺癌的二线治疗，但目前国内尚未获批适应证。

#### 3.1.2 免疫治疗

程序性死亡受体1（programmed cell death 1, PD-1）/程序性死亡配体1（programmed cell death ligand 1, PD-L1）抑制剂已成为驱动基因阴性NSCLC二线治疗新标准。基于III期CheckMate 078研究，国家药品监督管理局（National Medical Products Administration, NMPA）批准了纳武利尤单抗用于EGFR/间变性淋巴瘤激酶（anaplastic lymphoma kinase, ALK）阴性或未知IV期NSCLC的二线治疗，本共识予以一级推荐。CheckMate 078研究^[4]^是我国开展的首个以中国患者为主的PD-1抑制剂治疗晚期NSCLC的随机III期临床研究，结果显示，与多西他赛组相比，纳武利尤单抗组临床获益显著，ORR分别为17% vs 4%，中位OS分别为12.0 vs 9.5个月（HR=0.68, 97.7%CI: 0.52-0.90）。安全性数据显示，多西他赛组和纳武利尤单抗组总体治疗相关不良事件（treatment‐related adverse events, TRAEs）发生率分别为83%和64%，多西他赛治疗组的3-4级TRAEs发生率高于纳武利尤单抗组（47% vs 10%）。在RATIONALE 303的研究^[5]^中，替雷利珠单抗相较于多西他赛用于二线或三线治疗NSCLC临床获益显著，ORR为22.6% vs 7.1%，中位OS为17.2 vs 11.9个月（HR=0.64, P<0.0001）。在安全性方面，替雷利珠单抗相较于多西他赛≥3级TEAEs发生率更低。NMPA已经批准替雷利珠单抗用于EGFR/ALK阴性或未知的IV期NSCLC的二线治疗，本共识予以一级推荐。

### 3.2 免疫联合化疗经治患者二线治疗临床证据和推荐意见

**Table:** 

分层	一级推荐	二级推荐	三级推荐
PS 0-2分	多西他赛； 培美曲塞（非鳞癌） （如一线未使用同一药物）	多西他赛联合小分子抗血管生成药物安罗替尼（2A类）^[11]^；安罗替尼（不能耐受化疗）	参加临床研究

注：一部分研究考虑单臂小样本以及回顾性分析，证据级别较低，本共识作为注释部分，可供临床选择：雷莫西尤单抗联合多西他赛^[12]^、紫杉醇+贝伐珠单抗（非鳞癌）^[13]^。

针对于免疫经治患者的临床研究正在布局，但尚未有治疗方案在国内获批使用。考虑患者一线治疗对ICIs的反应及耐受性，后线研究探索时分为剔除、保留、换用ICIs等治疗方案：（1）剔除ICIs：采用抗血管生成药物联合化疗或者化疗；（2）保留原ICIs：原ICIs联合其他（放疗/化疗/抗血管生成药物/双免）；（3）换用ICIs：更换免疫治疗药物，可同时联合抗血管生成药物等治疗。本共识结合现有的免疫经治研究证据，基于专家投票为患者进行临床推荐。

对于PS评分0-2分患者一线免疫治疗进展后，可选择培美曲塞单药或多西他赛单药化疗（如果一线治疗未使用同一药物）。ALTER-L016与L018研究^[11]^共入组73例既往接受过ICIs治疗的NSCLC患者，均为中国患者，其中多西他赛联合安罗替尼组43例，多西他赛组30例，对这部分患者进行合并分析，结果显示，多西他赛联合安罗替尼组与多西他赛组比较，中位PFS分别为7.60和2.50个月（HR=0.28, P<0.0001）。本共识将安罗替尼联合多西他赛作为二级推荐。对于PS评分>2分的患者，建议最佳支持疗法。

另一项雷莫西尤单抗联合多西他赛对比多西他赛二线治疗经ICIs一线治疗失败的NSCLC临床研究^[12]^纳入39例既往接受过免疫治疗的患者，结果显示，联合组中位PFS为5.9个月，单药组为2.8个月（HR=0.75, P=0.03），中位OS分别为19.8 vs 8.6个月（P=0.10）。回顾性队列研究AVATAX^[13]^纳入314例转移性非鳞NSCLC患者，接受紫杉醇联合贝伐珠单抗用于二线及以上治疗。亚组分析显示，先期ICIs治疗的88例患者，二线治疗的中位PFS为7.0个月，中位OS为13个月。考虑到为回顾性研究亚组分析，在本共识中写入文字注释部分。

### 3.3 免疫单药经治患者二线治疗临床证据和推荐意见

**Table:** 

	推荐
一线免疫单药治疗（PD-L1 TPS≥50%）	含铂双药化疗

针对一线免疫单药经治患者[PD-L1肿瘤细胞阳性比例评分（tumor proportion score, TPS）≥50%]，目前二线常规推荐含铂双药化疗。进展后三线可选择多西他赛或培美曲塞（非鳞癌）单药化疗作为一级推荐。安慰剂对照、随机、多中心III期临床试验（ALTER 0303研究）^[14]^旨在探讨安罗替尼对二线及以上治疗后进展的NSCLC患者OS的影响。研究纳入606例经组织学或细胞学证实的NSCLC患者，有空洞的中央型鳞状细胞癌或脑转移灶未控制或控制不到2个月的患者被排除并以2:1的比例被随机分组，与安慰剂组相比，安罗替尼组中位OS显著延长（9.6 vs 6.3个月，HR=0.68，P=0.002），中位PFS显著延长（5.4 vs 1.4个月，HR=0.25，P<0.001），ORR显著提高[27 (9.2%) vs 1 (0.7%), P<0.001]。两组DCR间差异也具有统计学意义[238 (81.0%) vs 53 (37.1%), P<0.001]。

在驱动基因阴性晚期NSCLC患者中，安罗替尼可作为三线及以上治疗的一级推荐。另外，参加临床研究作为三线治疗患者的三级推荐。

## 4 未来研究方向展望

随着越来越多的ICIs在国内陆续获批上市，驱动基因阴性NSCLC患者的治疗已进入免疫治疗时代，部分III期临床研究^[3⇓-5]^显示，ICIs单药对比多西他赛二线治疗NSCLC能够显著延长OS。随着一线治疗格局的改变，免疫耐药人群急剧增加，二线治疗需要更多的研究进行探索，本共识介绍了中国临床肿瘤学会认可的常用治疗方式，包括抗血管生成药物联合化疗以及ICIs联合化疗。

除了以上常见的联合方式，更多的联合方式和创新药物研究正在探索当中，并且取得了初步的结果。Lung-MAP S1400I研究^[15]^纳武利尤单抗联合伊匹木单抗双免对比纳武利尤单抗单药治疗未接受过免疫治疗的经治肺鳞癌患者的效果，结果显示PFS和OS未能进一步获益。CONTACT-01研究^[16]^是一项探索阿替利珠单抗联合卡博替尼对比多西他赛单药治疗既往接受过ICIs和化疗的转移性NSCLC的疗效和安全性的III期研究，主要终点OS未达到。VARGADO研究队列C^[17]^中，多西他赛联合尼达尼布治疗免疫经治肺腺癌患者的中位PFS为4.8个月，ORR为37.5%。目前有几项关键的III期研究正在进行中。IFCT-1103 ULTIMATE研究^[18]^纳入166例化疗经治晚期非鳞NSCLC患者，结果证明，紫杉醇联合贝伐珠单抗组中位PFS为5.4个月，多西他赛组为3.9个月，结果具有统计学差异（HR=0.61, P=0.005）。KEYNOTE-010研究^[19]^显示，与多西他赛组比较，帕博利珠单抗2 mg/kg组、10 mg/kg组中位OS均得到改善（10.4 vs 8.5个月，HR=0.71，P=0.0008；12.7 vs 8.5个月，HR=0.61，P<0.0001）。OAK研究中^[20,21]^，相较于多西他赛组，阿替利珠单抗组主要研究终点中位OS显著延长（13.8 vs 9.6个月）（HR=0.73, P=0.0003），更新的4年OS率分别为15.5% vs 8.7%。在ORIENT-3研究^[22]^中，在经一线含铂化疗治疗失败的晚期鳞状NSCLC中，与多西他赛组相比，信迪利单抗治疗组中位OS（11.79 vs 8.25个月，HR=0.74，P=0.025）及PFS（4.30 vs 2.79个月，HR=0.52，P<0.001）均显著延长。TORG1630研究^[23]^是一项随机、多中心的II/III期临床试验，旨在对比纳武利尤单抗组和多西他赛联合纳武利尤单抗组用于经治晚期或复发性NSCLC患者的疗效，研究纳入128例既往均未接受过免疫治疗的患者，结果显示，单药治疗组与联合治疗组中位OS分别为14.7和23.1个月（HR=0.63, P=0.03）。免疫联合化疗中，一项多西他赛联合信迪利单抗在经治晚期NSCLC患者中的II期单臂临床研究^[24]^显示，中位PFS为5.78个月，中位OS为12.62个月，ORR为32.43%。PROLUNG研究^[25]^比较了多西他赛联合帕博利珠单抗与多西他赛单药二线治疗NSCLC的疗效，化疗联合免疫组与多西他赛单药组的ORR分别为42.5% vs 15.8%（OR=3.94, 95%CI: 1.34-11.5, P=0.01），两组中位PFS分别为9.5（95%CI: 4.2-NR） vs 3.9个月（95%CI: 3.2-5.7）（HR=0.24, 95%CI: 0.13-0.46, P<0.001）。

另外，有关新型治疗方式，LUNAR研究^[26]^探究肿瘤电场治疗联合标准治疗与单独使用标准治疗用于铂类药物治疗后进展的转移性NSCLC的疗效比较，研究达到了主要研究终点，其中，中位OS延长了3个月，具有统计学获益和临床获益（HR=0.74, P=0.035），肿瘤电场治疗耐受性良好，没有增加全身毒性，很少有3级（无4或5级）设备相关皮肤AEs。INSIGNA研究（NCT03793179）^[27]^纳入晚期非鳞且PD-L1 TPS≥1%的NSCLC患者，探索帕博利珠单抗治疗进展后二线帕博利珠单抗不同联合方式的疗效及安全性，研究正在进行中，结果值得期待。

探讨最佳的联合治疗方案是未来重要的研究方向，目前，抗血管生成药物联合治疗及ICIs联合治疗是临床治疗驱动基因阴性NSCLC二线治疗的重要策略，应用前景广阔，希望将来有更多的III期临床试验证据来验证这些联合治疗的可行性。

**Competing interests**

The authors declare that they have no competing interests.

**Table:** 

共识编写审定专家组成员	
**组长**	
周彩存	上海市东方医院
**副组长**	
王洁	中国医学科学院肿瘤医院
程颖	吉林省肿瘤医院
王宝成	解放军第九六〇医院
周清华	四川大学华西医院
**执笔专家**	
周斐	上海市肺科医院
**专家组成员**（按姓氏汉语拼音排名）	
段建春	中国医学科学院肿瘤医院/
	中国医学科学院肿瘤医院山西医院
范云	浙江省肿瘤医院
黄鼎智	天津医科大学肿瘤医院
林根	福建省肿瘤医院
柳江	新疆维吾尔自治区人民医院
柳影	吉林省肿瘤医院
潘跃银	中国科学技术大学附属第一医院
盛立军	山东第一医科大学第三附属医院
史美祺	江苏省肿瘤医院
宋勇	南京大学医学院附属金陵医院
王俊	山东第一医科大学第一附属医院
王启鸣	郑州大学附属肿瘤医院（河南省肿瘤医院）
邬麟	湖南省肿瘤医院（中南大学湘雅医学院
	附属肿瘤医院）
姚煜	西安交通大学第一附属医院
庄莉	云南省肿瘤医院（昆明医科大学第三附属医院）
